# Association of cerebrospinal inflammatory profile with radiological features in newly diagnosed treatment-naïve patients with multiple sclerosis

**DOI:** 10.3389/fneur.2022.1012857

**Published:** 2022-09-20

**Authors:** Shinji Ashida, Takayuki Kondo, Chihiro Fujii, Mio Hamatani, Toshiki Mizuno, Hirofumi Ochi

**Affiliations:** ^1^Department of Neurology, Graduate School of Medical Science, Kyoto Prefectural University of Medicine, Kyoto, Japan; ^2^Department of Neurology, Kansai Medical University Medical Center, Osaka, Japan; ^3^Institute for the Advanced Study of Human Biology, Kyoto University, Kyoto, Japan; ^4^Department of Intractable Disease and Aging Science, Ehime University Graduate School of Medicine, Toon, Japan

**Keywords:** multiple sclerosis, MRI, cerebrospinal fluid, cytokine, chemokine

## Abstract

**Objective:**

Multiple sclerosis (MS) is an immune-mediated demyelinating disease of the central nervous system. Without reliable diagnostic biomarkers, the clinical and radiological heterogeneity of MS makes diagnosis difficult. Although magnetic resonance imaging (MRI) is a major diagnostic tool for MS, the association of MRI findings with the inflammatory profile in cerebrospinal fluid (CSF) has been insufficiently investigated. Therefore, we focused on CSF profile of MS patients and examined its association with MRI findings.

**Methods:**

Concentrations of 26 cytokines and chemokines were determined in CSF of 28 treatment-naïve MS patients and 12 disease-control patients with aquaporin-4 antibody-seropositive neuromyelitis optica spectrum disorder (NMOSD).

**Results:**

High levels of interleukin (IL)-6, IL-17A, B-cell activating factor (BAFF), a proliferation inducing ligand (APRIL), and CD40 ligand were correlated with the absence of at least one of the following three MRI findings in MS: an ovoid lesion, three or more periventricular lesions, and a nodular and/or ring-shaped contrast-enhancing lesion. The multivariate analysis revealed that elevated IL-17A was an independent predictor of absence of ovoid lesion and periventricular lesions less than three. MS patients were classified into a group with all three MRI findings (MS-full) and a group with less than three (MS-partial). The discriminant analysis model distinguished three groups: MS-full, MS-partial, and NMOSD, with 98% accuracy.

**Conclusion:**

The CSF inflammatory profile was associated with radiological findings of treatment-naïve MS. This result indicates the possible utility of combined CSF and MRI profiling in identifying different MS phenotypes related to the heterogeneity of underlying immune processes.

## Introduction

Multiple sclerosis (MS) is an immune-mediated demyelinating disease of the central nervous system (CNS) (1). The diagnosis of MS is based on demonstration of the spatial and temporal occurrence of inflammatory demyelinating lesions, which is shown by magnetic resonance imaging (MRI) together with clinical presentation (2, 3). However, the diagnosis of MS is complex due to the marked heterogeneity in clinical manifestations and MRI features of lesions. Furthermore, the identification of 4 different immunopathological subtypes of MS has led to the hypothesis of intra-individual pathological homogeneity and inter-individual heterogeneity (4). There is no specific test currently available; MS is still diagnosed on the basis of clinical findings with supportive paraclinical evidence.

The most common laboratory finding supporting the diagnosis of MS is the detection of intrathecal synthesis of immunoglobulin G, most typically the presence of oligoclonal IgG bands (OCB) in the cerebrospinal fluid (CSF) (5). In addition, the analysis of CSF proteins provides useful information suggesting inflammatory processes within CNS. In this context, cytokines and chemokines play an important role in the evolution of MS lesions, and several pro-inflammatory cytokines and chemokines have been investigated as potential biomarkers of MS activity (6, 7). A recent study that combined CSF protein and MRI profiling revealed that high levels of pro-inflammatory CSF cytokine and chemokine molecules were associated with a higher cortical lesion load at MS diagnosis (8). These indicate that CSF parameters reflecting the heterogeneity of mechanisms involved in MS could help to predict disability outcomes and subsequently select the most appropriate therapy for individual MS patients. However, the association of CSF cytokine and chemokine profiles with MRI findings, such as the typical lesion morphology and characteristic distribution of lesions, which can support the diagnosis of MS, has yet to be investigated.

In this study, we analyzed the association of CSF cytokine and chemokine profiles with MRI findings in newly diagnosed treatment-naïve MS patients compared with control patients with aquaporin-4 (AQP4) antibody-seropositive neuromyelitis optica spectrum disorder (NMOSD), who underwent lumbar puncture in their diagnostic work-up.

## Materials and methods

### Patients

We included 29 consecutive newly diagnosed disease-modifying drug (DMD)-naïve relapsing-remitting MS patients and 12 consecutive disease-control patients with AQP4 antibody-seropositive NMOSD, in whom CSF samples were obtained in the diagnostic work-up at Kyoto Prefectural University of Medicine from August 2008 to August 2020. All MS patients fulfilled the McDonald 2010 criteria (9) and showed no radiological features suggestive of NMOSD, which include lesions in the peri-ependymal regions surrounding the lateral ventricles, diencephalon, periaqueductal region, area postrema, corticospinal tracts, extensive white matter involvement, optic chiasm, bilateral optic nerve, or longitudinally extensive spinal cord involvement (10, 11). One MS patient was excluded because the antibody test for myelin oligodendrocyte glycoprotein (MOG) was not performed on serum (12). Thus, the remaining 28 consecutive MS patients were included in this study and all of them were seronegative for both AQP4 and MOG antibodies. The clinical data including patients' demographic information and radiological findings at the time of CSF sampling were reviewed by neurologists (C.F. and S.A.). Physical disability was determined by the Expanded Disability Status Scale (EDSS). The disease duration was defined as the term from first episode with obvious neurological deficit to sample collection.

### Sample collection and flow cytometric analysis

Fresh CSF samples were taken from the MS and NMOSD patients in the acute phase and stored at −80°C in a freezer after being centrifuged. The acute phase was defined as the period within 28 days after neurological exacerbation; this exacerbation indicates neurological episodes lasting for more than 24 h. Cytokines and chemokines were measured using LEGENDplex^TM^ Human B Cell Panel [tumor necrosis factor (TNF)-α, interleukin (IL)-3, IL-4, IL-10, IL-6, IL-2, TNF-β, interferon (IFN)-γ, IL-17A, IL-12p70, a proliferation inducing ligand (APRIL), B-cell activating factor (BAFF), CD40L] and Human Proinflammatory Chemokine Panel 1 {C-X-C motif ligand (CXCL) 8 (IL-8), CXCL10 (IP-10), CC chemokine ligand (CCL) 11 (Eotaxin), CCL17 (thymus and activation-regulated chemokine: TARC), CCL5 (regulated on activation, normal T cell expressed and secreted: RANTES), CCL3 [macrophage Inflammatory Protein (MIP)-1 α: MIP-1α], CXCL9 (monokine induced gamma interferon: MIG), CCL20 (MIP-3α), CXCL1 (growth-regulated oncogene α: GROα), CXCL11 (interferon-inducible T-cell alpha chemoattractant: I-TAC), CCL4 (MIP-1β), and CCL2 (monocyte chemoattractant protein-1: MCP-1)}. All protocols followed the manufacturers' instructions and data were acquired using a FACS Canto II flow cytometer (BD Biosciences, Franklin Lakes, NJ, USA).

### Ethics

This study was approved by the Medical Ethics Committee of Kyoto Prefectural University of Medicine according to the tenets of the Declaration of Helsinki. Using an opt-out approach, we provided information on the research, including the purpose, and guaranteed that patients could request exclusion. All subjects provided written informed consent.

### Statistical analysis

The Expanded Disability Status Scale (EDSS) is shown as the median and range (25–75%). Other data are presented as the mean ± standard deviation (SD). Statistical analysis was conducted using the Mann–Whitney U test for analysis of unpaired data. The linear regression analysis was achieved to assess the relation between CSF cytokine/chemokine levels and age, EDSS, and disease duration. The multiple logistic regression was used to predict radiological features using CSF profile. Correlation rank was evaluated by Spearman's rank correlation tests. Discriminant analysis was used to determine groups, and explanatory variables were determined using the stepwise selection method. All data-sets were analyzed using R package (Ver. 4.2.0, R foundation for Statistical Computing, Vienna, Austria) and JMP^®^ 13 (SAS Institute Inc., Cary, NC, USA). A *p* < 0.05 was considered significant.

## Results

### Demographic characteristics, laboratory and radiological findings

We identified 28 consecutive newly diagnosed DMD-naïve relapsing-remitting MS patients and 12 consecutive AQP4 antibody-seropositive NMOSD patients. First, we analyzed demographic characteristics of the two groups (Table 1). The mean age at enrollment was younger in MS than in NMOSD patients (40.9 ± 12.2 vs. 57.7 ± 13.9 years, respectively, *p* = 0.0012). Twenty-three (82%) of the MS patients and 8 (75%) of the NMOSD patients were female. The mean disease duration was 2.5 ± 2.7 and 3.7 ± 7.5 years in MS and NMOSD patients, respectively. Neurological deficits evaluated by EDSS at the time of CSF sampling were higher in NMOSD than MS patients [4.3 (2.6–8.4) vs. 2.0 (1.0–2.9), respectively, *p* = 0.002]. There was no difference in the duration from onset of relapse to sample collection between MS and NMOSD patients (14.7 ± 10.2 vs. 14.7 ± 12.1 days, respectively, *p* = 0.78). The serum anti-nuclear antibody was detected in 3 of 12 NMOSD patients, but not in MS (25 vs. 0%, p = 0.006).

**Table 1:** Demographic characteristics, laboratory and radiological features.

	**MS (*n* = 28)**	**NMOSD (*n* = 12)**	***p* value**
**Demographics**			
Age (mean ± SD)	40.9 ± 12.2	57.7 ± 13.9	**0.0012**
Female	23 (82%)	8 (75%)	0.28
Age at onset (mean ± SD)	38.4 ± 12.7	53.9 ± 18.4	**0.012**
Disease duration (years) (mean ± SD)	2.5 ± 2.7	3.7 ± 7.5	0.27
Mean EDSS (range)	2.0 (1.0–2.9)	4.3 (2.6–8.4)	**0.002**
Relapse to sample collection (days) (mean ± SD)	14.7 ± 10.2	14.7 ± 12.1	0.78
Serum anti-nuclear antibody (>1:640 dilutions)	0	3 (25%)	**0.006**
**CSF findings**			
CSF cell count (cells/μl)	9.3 ± 17.3	6.9 ± 9.1	0.96
CSF protein (mg/dl)	37.9 ± 17.7	41.1 ± 13.2	0.19
Positive CSF	17/28 (61%)	2/9 (33%)	**0.04**
IgG index >0.7	3/28 (11%)	1/9 (11%)	0.97
Positive OCB	15/28 (54%)	1/6 (17%)	0.43
**Radiological findings**			
Subcortical lesions	20 (71%)	2 (17%)	**0.001**
PV lesions ≥3	22 (79%)	3 (25%)	**0.001**
Callosal septal lesions	16 (57%)	5 (42%)	0.37
Deep gray matter lesions	1 (4%)	1 (8%)	0.54
Infratentorial lesions	16 (57%)	3 (25%)	0.06
Spinal cord lesions	19 (68%)	7 (58%)	0.56
Ovoid lesions	20 (71%)	1 (8%)	**0.0001**
Periventricular radial lesions (Dawson's finger)	19 (68%)	4 (33%)	**0.04**
Nodular- and/or ring-shaped contrast-enhancing lesions	5/10 (50%)	0	NaN

MS, multiple sclerosis; NMOSD, neuromyelitis optica spectrum disorder; SD, standard deviation; EDSS, expanded disability status scale; CSF, central spinal fluid; OCB, oligoclonal bands; DGM, deep gray matter; PV, periventricular lesion; CE, contrast-enhancing; NaN, not a number. Bold values denote statistical significance at the *p* < 0.05 level.

A positive CSF study, defined as showing either OCB or a high IgG index (>0.7) (13), was more common in MS than NMOSD patients (61 vs. 33%, respectively, *p* = 0.04). Other components of CSF analysis, such as the cell count and protein, did not differ significantly between the two groups.

Next, we assessed radiological features, such as the lesion location, morphology, and contrast-enhancing shape, between MS and NMOSD patients. Subcortical lesions were more common in MS than NMOSD patients (71 vs. 36%, respectively, *p* = 0.001). The presence of more than three periventricular lesions (PV ≥ 3) was significantly more common in MS than NMOSD patients (79 vs. 25%, respectively, *p* = 0.001). Ovoid and periventricular radial lesions (Dawson's fingers) were also significantly more common in MS than NMOSD patients (71 vs. 8%, *p* = 0.0001, and 68 vs. 33%, *p* = 0.04, respectively). Contrast-enhancing (CE) lesions were noted in 10 of the 28 MS patients, but not in NMOSD patients (36 vs. 0%, respectively, *p* = 0.02). Nodular and/or ring-shaped contrast-enhancing lesions, which are typical in MS, were noted in 5 of the 10 (50%) MS patients with contrast-enhancing lesions.

### Correlation of CSF inflammatory profile with radiological findings at MS diagnosis

**Figure 1:**
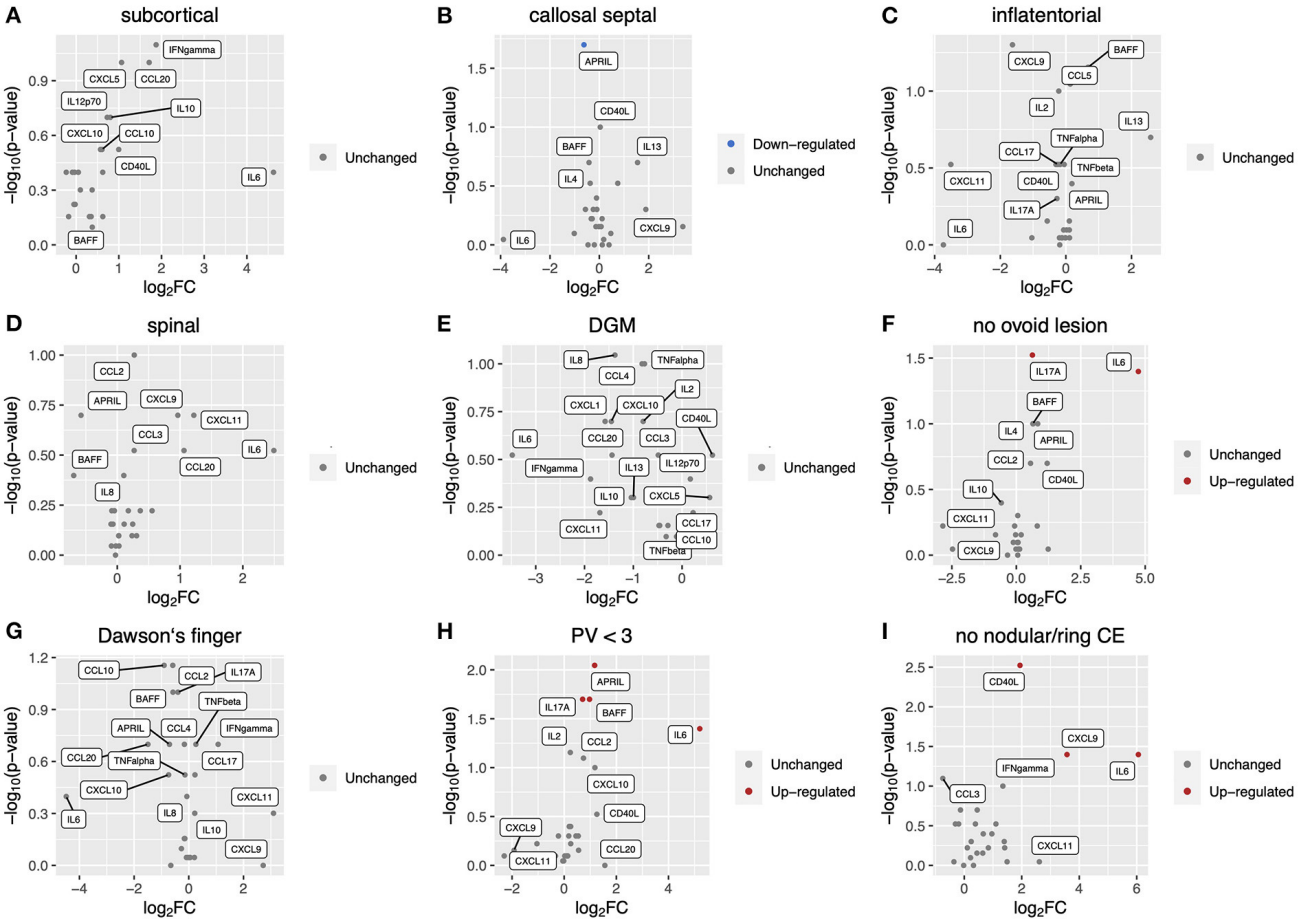
Correlation of CSF inflammatory profile with radiological findings at MS diagnosis. Volcano plot showing log2 (fold change, FC) against –log10 (*p*-value) of CSF inflammatory profile based on radiological findings as follows: **(A)**, subcortical lesion; **(B)**, callosal septal lesion; **(C)**, inflatentorial lesion; **(D)**, spinal lesion; **(E)**, DGM lesion; **(F)**, no ovoid lesion; **(G)**, Dawson‘s finger; **(H)**, less than three PV. Red or blue indicates elevation or reduction, respectively, of cytokine/chemokine levels with a significant FC (|FC| > 1.5) and p-value (< 0.05). CSF, cerebrospinal fluid; MS, multiple sclerosis; DGM, deep gray matter lesion; PV, periventricular lesions; CE, contrast-enhancing.

**Figure 2:**
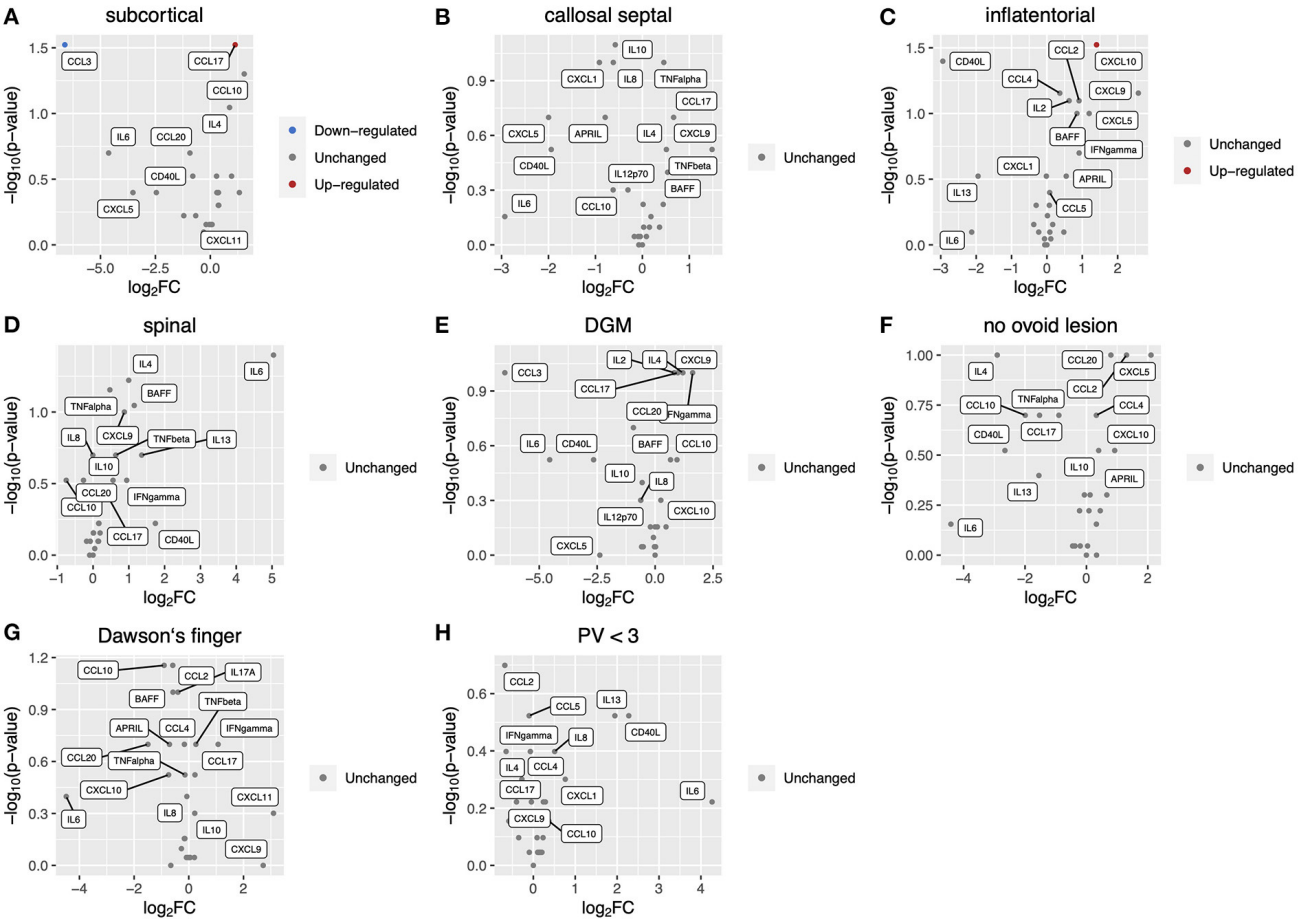
Correlation of CSF inflammatory profile with radiological findings in NMOSD patients. Volcano plot showing log2 (fold change, FC) against –log10 (*p*-value) of CSF inflammatory profile based on radiological findings as follows: **(A)**, subcortical lesion; **(B)**, callosal septal lesion; **(C)**, inflatentorial lesion; **(D)**, spinal lesion; **(E)**, DGM lesion; **(F)**, no ovoid lesion; **(G)**, Dawson's finger; **(H)**, less than three PV. Red or blue indicates elevation or reduction, respectively, of cytokine/chemokine levels with a significant FC (|FC| > 1.5) and *p*-value (<0.05). CSF, cerebrospinal fluid; NMOSD, neuromyelitis optica spectrum disorder; DGM, deep gray matter lesion; PV, periventricular lesions; CE, contrast-enhancing.

We measured levels of 26 key cytokines and chemokines in CSF and analyzed the association of the CSF profile with radiological findings of MS at diagnosis (Figures 1A–I). The absence of at least one of three radiological findings typical of MS: an ovoid lesion, PV ≥ 3, and a nodular and/or ring-shaped contrast-enhancing (typical CE) lesion, was significantly more common in patients with high levels of certain cytokines and chemokines in CSF. The IL-6 level was significantly higher in patients without an ovoid lesion, PV ≥ 3, or a typical CE lesion (*p* = 0.04, 0.04, and 0.04, respectively) (Figures 1F,H,I). The level of IL-17A was elevated in patients without an ovoid lesion or PV ≥ 3 (*p* = 0.03 and 0.02, respectively) (Figures 1F,H). APRIL and BAFF were significantly higher in patients without PV ≥ 3 (*p* = 0.009 and 0.02, respectively) (Figure 1H). On the contrary, APRIL was lower in patients with a callosal septal lesion (*p* = 0.02) (Figure 1B). CD40L and CXCL9 levels were higher in patients without than with a typical CE lesion (*p* = 0.003 and 0.04, respectively) (Figure 1I). These indicate that the CSF molecular profile was associated with radiological findings of MS. Of interest, except for a callosal septal lesion, which belongs to a PV lesion, all three radiological findings associated with the CSF molecular profile have been reported to distinguish between MS and NMOSD (14, 15). In addition, we recently reported that the absence of these radiological findings was associated with corticosteroid and/or immunosuppressant (CS/IS) use for preventative treatment in patients with an established diagnosis of MS (16). We also compared the CSF profile with radiological findings of NMOSD (Figures 2A–H). There was no association of the CSF molecular profile with radiological findings of NMOSD determined by the presence or absence of an ovoid lesion and PV ≥ 3 (Figures 2F,H). None of the NMOSD patients showed CE lesions.

We performed multivariate analysis of CSF profile which showed significant change between MS patients with and without an ovoid lesion, PV ≥ 3, or a typical CE lesion (Table 2). The multivariate analysis revealed that IL-17A elevation was an independent predictor of radiological findings: lack of ovoid lesion and PV <3 [odds ratio = 2.52 (1.12–5.17), *p* = 0.0049 and odds ratio = 2.24 (1.03–4.87), *p* = 0.015, respectively].

**Table 2:** Multivariate analysis of radiological findings among MS patients using cytokine/chemokine levels in CSF.

**Cytokine/Chemokine**	**Standardized partial regression coefficient**	**Odds ratio**	**95% confidence interval**	***p***
**Lack of ovoid lesion**				
IL-17A	0.88	2.52	1.12–5.17	**0.0049**
APRIL	−0.0058	0.99	0.99–1.00	0.18
CXCL9	−0.14	0.87	0.66–1.16	0.2
BAFF	0.0019	1	0.99–1.01	0.23
IL-6	0.0018	1	0.99–1.00	0.48
CD40L	0.053	1.05	0.88–1.27	0.57
**PV** **<** **3**				
IL-17A	0.81	2.24	1.03–4.87	**0.015**
CXCL9	−0.19	0.82	0.52–1.30	0.21
BAFF	0.0014	1	0.99–1.00	0.38
IL-6	0.0023	1	0.99–1.00	0.4
APRIL	−0.0035	1	0.99–1.00	0.42
CD40L	0.03	1	0.85–1.25	0.76
**No nodular/ring CE**				
CXCL9	0.144	1.15	0.96–1.39	0.1
CD40L	0.173	1.18	0.93–1.51	0.14
IL-6	−0.0000767	1	0.99–1.00	0.47
BAFF	0.00166	1	0.99–1.00	0.5
IL-17A	0.217	1.24	0.46–3.34	0.65
APRIL	−0.000197	1	0.99–1.01	0.75

MS, multiple sclerosis; PV, periventricular lesion; CE, contrast-enhancing. Bold values denote statistical significance at the *p* < 0.05 level.

### Two MS subgroups with different radiological findings associated with CSF profile

MS patients were classified into two groups according to three radiological findings associated with the CSF profile; patients who showed all three findings were classified into the MS-full group, and the others into the MS-partial group. Among the 28 MS patients, 17 (61%) were classified into the MS-full group and the others (39%) into the MS-partial group (Table 3).

**Table 3:** Demographic features between MS-full and MS-partial groups.

	**MS-full (*n* = 17)**	**MS-partial (*n* = 11)**	***p* value**
**Demographics**			
Age (mean ± SD)	39.3 ± 11.9	43.5 ± 12.7	0.37
Female	16 (94%)	7 (64%)	**0.048**
Age at onset (mean ± SD)	35.9 ± 12.1	42.3 ± 13.3	0.17
Disease duration (years) (mean ± SD)	3.4 ± 3.0	1.2 ± 1.7	**0.048**
EDSS (range)	1.5 (1.0-2.3)	2.5 (2.0-3.5)	**0.008**
ARR at enrollment	1.0 ± 0.7	1.6 ± 1.1	0.14
Relapse to sample collection (days) (mean ± SD)	13.4 ± 10.5	16.7 ± 10.1	0.32
Serum autoantibody (>1:640 dilutions)	0	0	NaN
**CSF findings**			
CSF cell count (cells/μl)	3.2 ± 3.9	18.5 ± 25.1	**0.02**
CSF cell count (>50 cells/μl)	0 (0%)	1 (9%)	
CSF protein (mg/dl)	31.9 ± 12.4	47.1 ± 21.0	**0.04**
CSF protein (>100 mg/dl)	0 (0%)	0 (0%)	
IgG index >0.7	3 (18%)	0 (0%)	0.1
OCB positivity	11 (65%)	4 (36%)	0.14
**Radiological findings**			
Number of T2 lesions (mean ± SD)	12.3 ± 5.9	8.6 ± 6.4	0.11
Presence of contrast-enhancing lesions	4 (24%)	6 (55%)	0.09
Subcortical lesions	13 (76%)	7 (64%)	0.47
PV lesions >3	17 (100%)	5 (45%)	**0.0002**
Callosal septal lesions	12 (71%)	4 (36%)	0.07
Deep gray matter lesions	0	1 (9%)	0.17
Infratentorial lesions	9 (53%)	7 (64%)	0.58
Spinal cord lesions	12 (71%)	7 (64%)	0.7
Ovoid lesions	17 (100%)	3 (27%)	**<0.0001**
Periventricular radial lesions (Dawson's finger)	14 (82%)	5 (45%)	**0.04**
Nodular- and/or ring-shaped contrast-enhancing lesions	4/4 (100%)	1/6 (17%)	**0.03**

MS, multiple sclerosis; NMOSD, neuromyelitis optica spectrum disorder; SD, standard deviation; EDSS, expanded disability status scale; ARR, annual relapse rate; CSF, cerebrospinal fluid; OCB, oligoclonal bands; PV, periventricular lesion; NaN, not a number. Bold values denote statistical significance at the *p* < 0.05 level.

The mean age at enrollment was 39.3 ± 11.9 years in the MS-full group and 43.5 ± 12.7 years in the MS-partial group. Sixteen (94%) of the MS-full group and 7 (64%) of the MS-partial group were female, with marginal significance (*p* = 0.048). The disease duration was longer in the MS-full group than MS-partial group, with marginal significance (3.4 ± 3.0 vs. 1.2 ± 1.7 years, respectively, *p* = 0.048). EDSS was higher in the MS-partial group than MS-full group (2.5 vs. 1.3, respectively, *p* = 0.008). There was no significant correlation between CSF inflammatory parameters with age (Supplementary Figure 1), EDSS (Supplementary Figure 2) and disease duration (Supplementary Figure 3). The annual relapse rate at enrollment did not differ significantly between the two groups (1.0 ± 0.7 in MS-full vs. 1.6 ± 1.1 in MS-partial, *p* = 0.14). There was no difference in the duration from onset of relapse to sample collection between MS-full and MS-partial group (13.4 ± 10.5 vs. 16.7 ± 10.1 days, respectively, *p* = 0.32). The cell count and protein level in CSF were significantly higher in the MS-partial group than MS-full group (18.5 ± 25.1 vs. 3.2 ± 3.9 cells/μl, *p* = 0.02, and 47.1 ± 21.0 vs. 31.9 ± 12.4 mg/dl, *p* = 0.04, respectively). One patient in the MS-partial group showed a cell count of more than 50/μl in CSF. The OCB positivity rate did not differ significantly between the groups (65% in MS-full group and 36% in MS-partial group). Brain MRI activity based on the number of T2 lesions (12.3 ± 5.9 in MS-full group vs. 8.6 ± 6.4 in MS-partial group, *p* = 0.11) or identification of contrast-enhancing lesions (24% in MS-full group vs. 55% in MS-partial group, *p* = 0.09) did not differ significantly between the groups. Periventricular radial lesions were more common in the MS-full group than MS-partial group (82 vs. 45%, respectively, *p* = 0.04). The frequency of all other radiological findings, except for the three radiological findings associated with the CSF profile, did not differ significantly between the groups.

### Correlation of cytokine and chemokine levels in CSF

**Figure 3:**
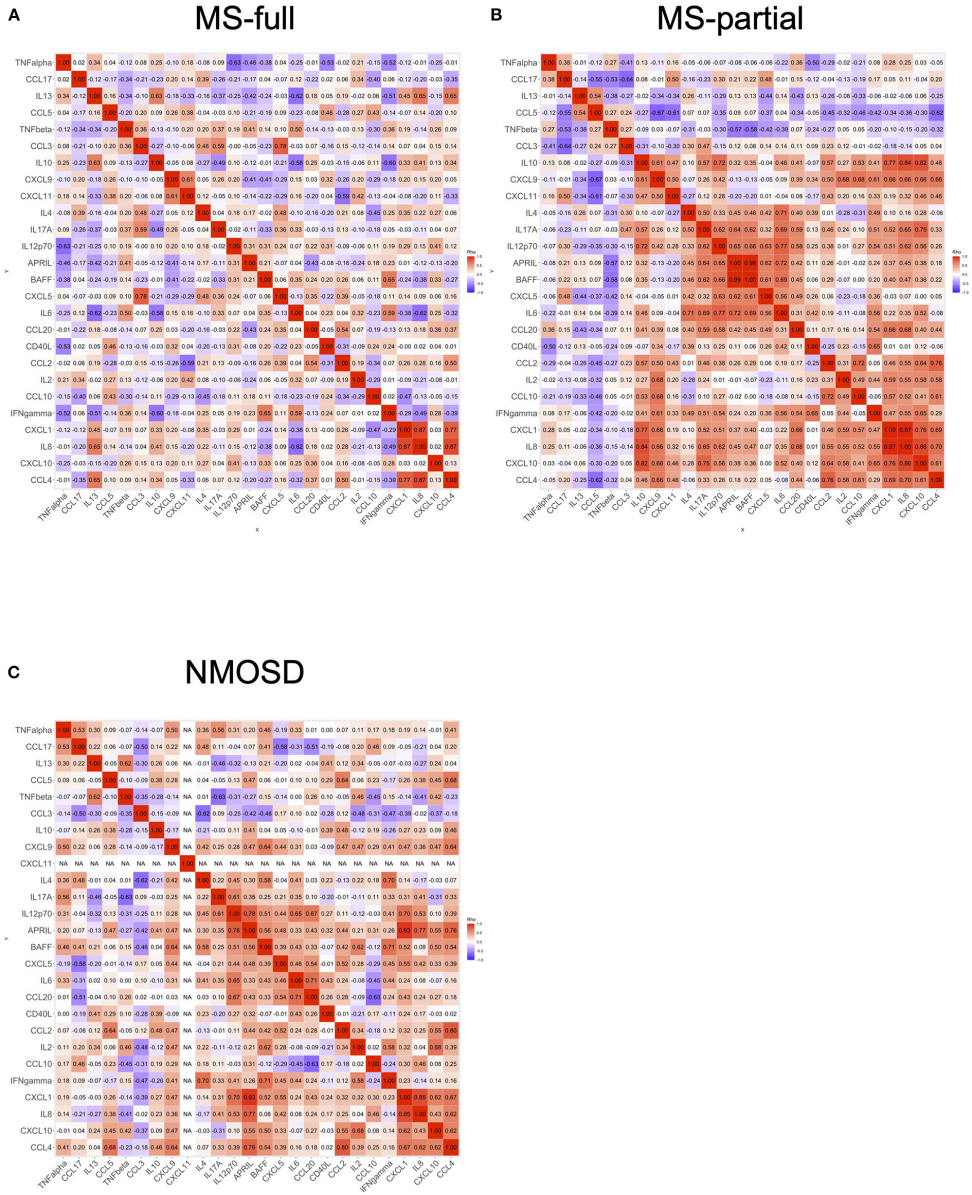
Correlation of cytokine and chemokine levels in CSF. The correlation of cytokine and chemokine levels in all MS and NMOSD groups. Correlation rank was evaluated by Spearman's rank correlation tests. Each heatmap showed the correlation between the levels of cytokines or chemokines: MS-full **(A)**, MS-partial **(B)**, and NMOSD **(C)**. CSF, cerebrospinal fluid; MS, multiple sclerosis; NMOSD, neuromyelitis optica spectrum disorder.

We analyzed the correlation of cytokine and chemokine levels in all MS and NMOSD groups. A heatmap showed the correlation between levels of cytokines and chemokines in three groups (Figures 3A–C). Several clusters which indicated significant correlations were detected in the MS-partial group and NMOSD group, but not in the MS-full group (Figures 3A–C).

### Discriminant analysis using cytokine and chemokine levels in CSF

**Figure 4:**
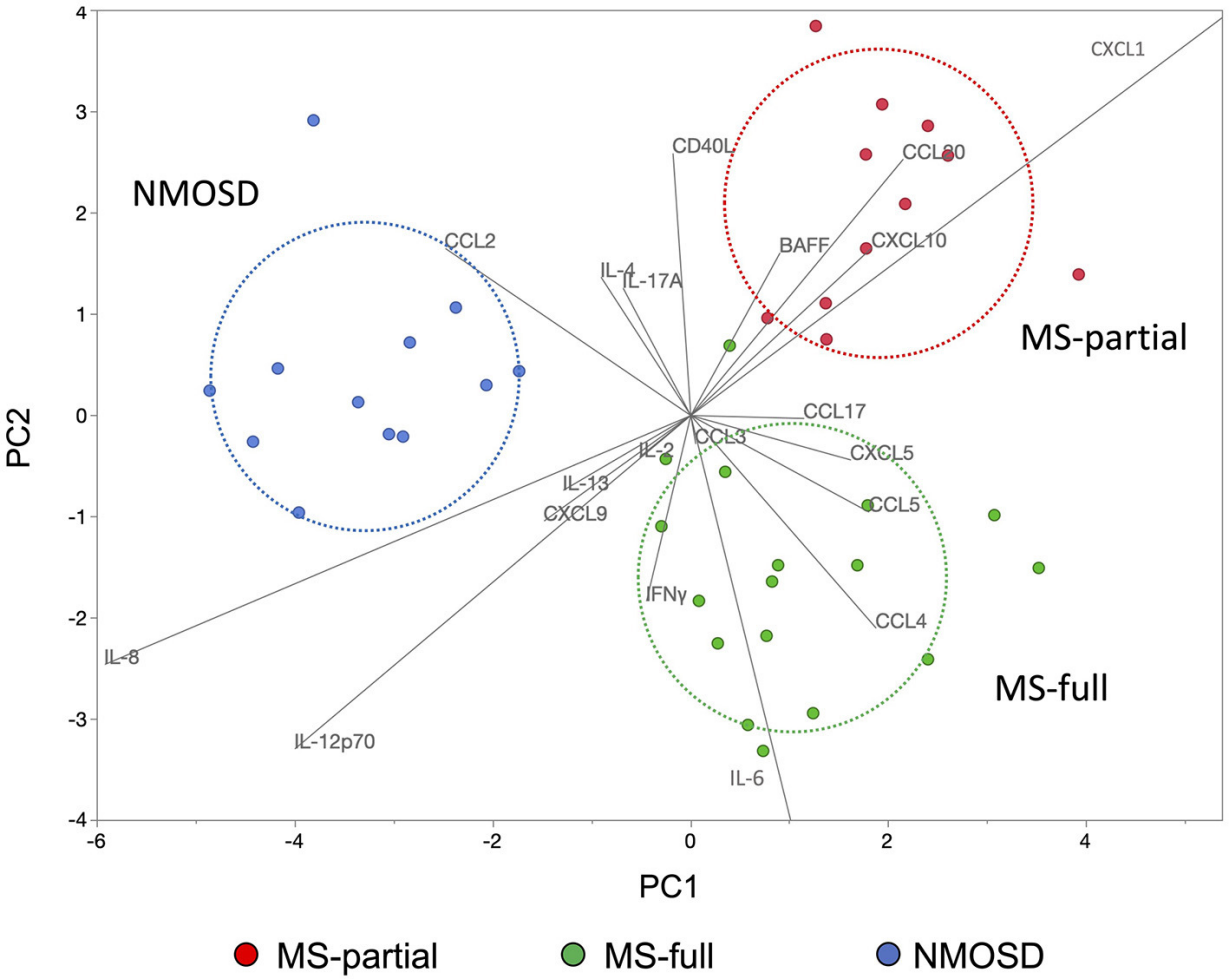
Discriminant analysis using cytokine and chemokine levels in CSF. The use of discriminant analysis to confirm that the characteristics of cytokine and chemokine levels in the CSF could be used to distinguish three groups: MS-full (green dots), MS-partial (red dots), and NMOSD (blue dots) groups. Explanatory variables were decided by the stepwise selection method. Each circle indicates the determined group area with 50% probability. CSF, cerebrospinal fluid; MS, multiple sclerosis; NMOSD, neuromyelitis optica spectrum disorder.

Discriminant analysis was performed to confirm whether the characteristics of cytokine and chemokine levels in CSF could be used to distinguish among three groups: MS-full, MS-partial, and NMOSD groups. Explanatory variables (IL-17A, CCL3, CCL2, CXCL10, CXCL5, CCL5, CCL4, IFNγ, CD40L, IL-8, IL-6, IL-4, IL-2, CXCL9, IL-13, IL-12p70, CXCL1, BAFF, CCL20, and CCL17) were decided by a stepwise selection method (entropy *R*^2^ = 0.96). The discriminant analysis model is shown in Figure 4. This discriminant analysis model distinguished the three groups with 98% accuracy. One MS patient who showed all three radiological findings associated with the CSF profile was included in the MS-partial group. Each circle indicates the determined group area with 50% probability. The NMOSD group was well-determined and the distance between MS-partial and MS-full groups was shorter than that between MS-full and NMOSD groups. CCL4 and CCL5 were characteristic in the MS-full group-determined area. At the same time, skews of BAFF, CXCL10, and CCL20 levels were indicated in the MS-partial group-determined area. Retrospective analysis revealed that all patients in the MS-partial group were treated with CS/IS after being diagnosed with MS.

## Discussion

In this study, the CSF inflammatory profile was associated with radiological findings at MS diagnosis. The elevation of IL-6, IL-17A, APRIL, BAFF, and CD40L levels in CSF was associated with the absence of at least one of the following three radiological findings: 1: an ovoid lesion, 2: PV ≥ 3, and 3: a nodular and/or ring-shaped CE lesion, which are considered typical of MS in terms of the lesion morphology, distribution, and contrast-enhancement pattern on conventional MRI sequences (2, 3, 16, 17). The multivariate analysis revealed that high level of IL-17A in CSF was an independent predictor of absence of ovoid lesion and periventricular lesions less than three.

IL-6, IL-17A, APRIL, and BAFF in CSF were reported to be higher in NMOSD than MS (14, 15, 18, 19). In this study, the MS-partial group is on the borderline between MS-full and NMOSD groups in terms of demographic characteristics, such as the age at disease onset and physical disability evaluated by EDSS, and CSF inflammatory activity assessed by cell count and protein level. However, none of the patients in MS-partial group, in whom at least one of the three radiological findings were absent on MRI at MS diagnosis, were seropositive for both AQP4 and MOG antibodies. Furthermore, the MS-partial group shared radiological findings other than the three associated with the CSF inflammatory profile with the MS-full group.

Higher CSF inflammatory activity in MS-partial group might be associated with higher EDSS than MS-full group. This difference in CSF inflammatory activity between the two MS groups suggests that underlying immunological mechanisms differ among them. Indeed, correlation analysis of cytokine and chemokine levels in CSF revealed clearly different expression patterns between MS-full and MS-partial groups. Of importance, the discriminant model based on the CSF profile suggested that MS-full and MS-partial groups were completely different from the NMOSD group.

CCL4 and CCL5 were characteristic chemokines in the MS-full group, and BAFF and CXCL10 were pathognomonic in the MS-partial group. The elevation of CCL4 and CCL5 levels in CSF was reported in patients with MS (20, 21). A previous study revealed that the expression of CCL5 on the blood vessel endothelium, perivascular cells, and surrounding astrocytes was detected in actively demyelinating MS plaques (22). Also, CCL4 was expressed by macrophages and microglia present within the inflammatory MS lesion (22). Thus, these chemokines play a crucial role in the recruitment of T cells and mononuclear phagocytes into CNS inflammatory lesions from peripheral blood (22, 23). On the other hand, CXCL10 and BAFF are crucial for the B-cell immune response (14, 24). These immunological differences indicate that the CSF inflammatory profile reflects the heterogeneity of immune mechanisms involved in MS. All of MS patients enrolled in this study showed at least one relapse and were considered the introduction of prevention therapy. In this context, it is worthy of note that 16 of the 17 patients (94%) in the MS-full group received disease-modifying drugs for MS (MS-DMDs) and all patients in the MS-partial group received CS/IS for relapse prevention based on retrospective analysis. One patient in the MS-full group did not receive relapse prevention therapy. This observation was consistent with our previous report of an association of radiological features with the therapeutic choice in MS (16). However, it is sometimes difficult to select the most appropriate therapy for individual MS patients based on radiological findings at the point of diagnosed. Indeed, 18% of patients in MS-partial group received MS-DMDs initially and then were switched to CS/IS in this study. Similarly, 9% of patients initially treated with MS-DMDs were switched to CS/IS and 17% of patients initially treated with CS/IS were switched to MS-DMDs subsequently in our previous study (16). Therefore, combined profiling of inflammatory mediators in CSF and MRI findings at the point of diagnosis could further help in identifying different MS phenotypes related to the heterogeneity of underlying immunological mechanisms and consequently in selecting appropriate therapy for individual MS patients. However, a further prospective clinical study in another MS cohort is needed to clarify whether the combined CSF and MRI profiling is linked to an appropriate treatment choice.

This study had several limitations. First, this was a small study conducted at a single center. Second, this was a retrospective study, and CSF profiling should be assessed in another MS cohort to confirm that this model is useful for the selection of prevention therapy in MS patients. Third, three patients with MS (one in the MS-full group and two in the MS-partial group) and five patients with NMOSD received intravenous methylprednisolone (IVMP) therapy before undergoing lumbar puncture. IVMP therapy might influence cytokine and chemokine levels in CSF. However, all eight patients were included in each determined area with 50% probability. Forth, CSF samples were taken in different timing of acute period, but the duration from onset of relapse to sample collection did not differ significantly among MS-full, MS-partial, and NMOSD groups (13.4 ± 10.5, 16.7 ± 10.1, and 14.7 ± 12.1 days, respectively). Besides, there was no correlation between CSF inflammatory parameters with the duration of acute exacerbation at sample collection (Supplementary Figure 4).

In conclusion, the CSF inflammatory profile was associated with MRI findings of DMD-naïve MS patients at diagnosis and reflected the immunological heterogeneity. Analysis of the CSF profile might subsequently be useful for the decision on appropriate prevention therapy in MS, but a further clinical prospective study is needed to confirm the usefulness of the CSF analysis model.

## Data availability statement

The raw data supporting the conclusions of this article will be made available by the authors, without undue reservation.

## Ethics statement

The studies involving human participants were reviewed and approved by Medical Ethics Committee of Kyoto Prefectural University of Medicine. The patients/participants provided their written informed consent to participate in this study.

## Author contributions

SA designed this study, analyzed clinical and biological data, and contributed to writing the manuscript. TK assisted and oversaw experimental design and data interpretation and supervised the process of writing the manuscript. CF and MH assisted in experimental design and data interpretation and collected patients' clinical data and samples. TM assisted in data interpretation and writing the manuscript. HO assisted and oversaw experimental design and data interpretation and contributed to writing the manuscript. All authors contributed to the article and approved the submitted version.

## Funding

This research was funded by Grant-in-Aid for Research Activity for Scientific Research (C) (Grant Number JP 21K07463) (HO), Grant-in-Aid for Research Activity start-up (Grant Number JP 20K22786) (SA), and Grant-in-Aid for Early-Career Scientists (Grant Number JP 19K16923) (CF).

## Conflict of interest

Author SA received speaker honoraria from Novartis Pharma K.K., and Biogen Japan Ltd. Author CF received speaker honoraria from Biogen Japan Ltd., Novartis Pharma K.K., Mitsubishi Tanabe Pharma Corp., Alexion Pharmaceuticals Inc., Teijin Healthcare Ltd., Bayer Yakuhin Ltd., Daiichi Sankyo Co., Ltd., and Takeda Pharmaceutical Co., Ltd. Author HO was on the scientific advisory board for Biogen Japan Ltd., Novartis Pharma K.K., Mitsubishi Tanabe Pharma Corp., and Alexion Pharmaceuticals Inc., and received speaker honoraria from Biogen Japan Ltd., Novartis Pharma K.K., Mitsubishi Tanabe Pharma Corp., Alexion Pharmaceuticals Inc., Chugai Pharmaceutical Co., Ltd., Nihon Pharmaceutical Co., Ltd., and Takeda Pharmaceutical Co., Ltd.

The remaining authors declare that the research was conducted in the absence of any commercial or financial relationships that could be construed as a potential conflict of interest.

## Publisher's note

All claims expressed in this article are solely those of the authors and do not necessarily represent those of their affiliated organizations, or those of the publisher, the editors and the reviewers. Any product that may be evaluated in this article, or claim that may be made by its manufacturer, is not guaranteed or endorsed by the publisher.

## Supplementary material

Supplementary Figure 1 Correlation of CSF inflammatory parameters with age at sample collection. Scatterplot graphs of the linear regression analysis showed the relation between cytokine/chemokine levels in CSF and age at sample collection. Strengths of the associations are given in *R*^2^ values. CSF, cerebrospinal fluid; MS, multiple sclerosis; NMOSD, neuromyelitis optica spectrum disorder.

Supplementary Figure 2 Correlation of CSF inflammatory parameters with EDSS at sample collection. Scatterplot graphs of the linear regression analysis showed the relation between cytokine/chemokine levels in CSF and EDSS at sample collection. Strengths of the associations are given in *R*^2^ values. CSF, cerebrospinal fluid; MS, multiple sclerosis; NMOSD, neuromyelitis optica spectrum disorder; EDSS, Expanded Disability Status Scale.

Supplementary Figure 3 Correlation of CSF inflammatory parameters with disease duration at sample collection. Scatterplot graphs of the linear regression analysis showed the relation between cytokine/chemokine levels in CSF and DD at sample collection. Strengths of the associations are given in *R*^2^ values. The DD was defined as the term (year) from first episode with neurological deficit to sample collection. CSF, cerebrospinal fluid; MS, multiple sclerosis; NMOSD, neuromyelitis optica spectrum disorder; DD, disease duration.

Supplementary Figure 4 Correlation of CSF inflammatory parameters with duration of acute exacerbation at sample collection. Scatterplot graphs of the linear regression analysis showed the relation between cytokine/chemokine levels in CSF and ADD at sample collection. Strengths of the associations are given in *R*^2^ values. The ADD was defined as the term (day) from onset of acute neurological deficit to sample collection. CSF, cerebrospinal fluid; MS, multiple sclerosis; NMOSD, neuromyelitis optica spectrum disorder; ADD, acute disease duration.

## Abbreviations

MS: multiple sclerosis
NMOSD: neuromyelitis optica spectrum disorder
CSF: cerebrospinal fluid.
