# Supplementary figures and images for: Association of cerebrospinal inflammatory profile with radiological features in newly diagnosed treatment-naïve patients with multiple sclerosis
Source: Front Neurol. 2022 Sep 20;13:1012857. doi: 10.3389/fneur.2022.1012857 (PMC9530286; doi:10.3389/fneur.2022.1012857)

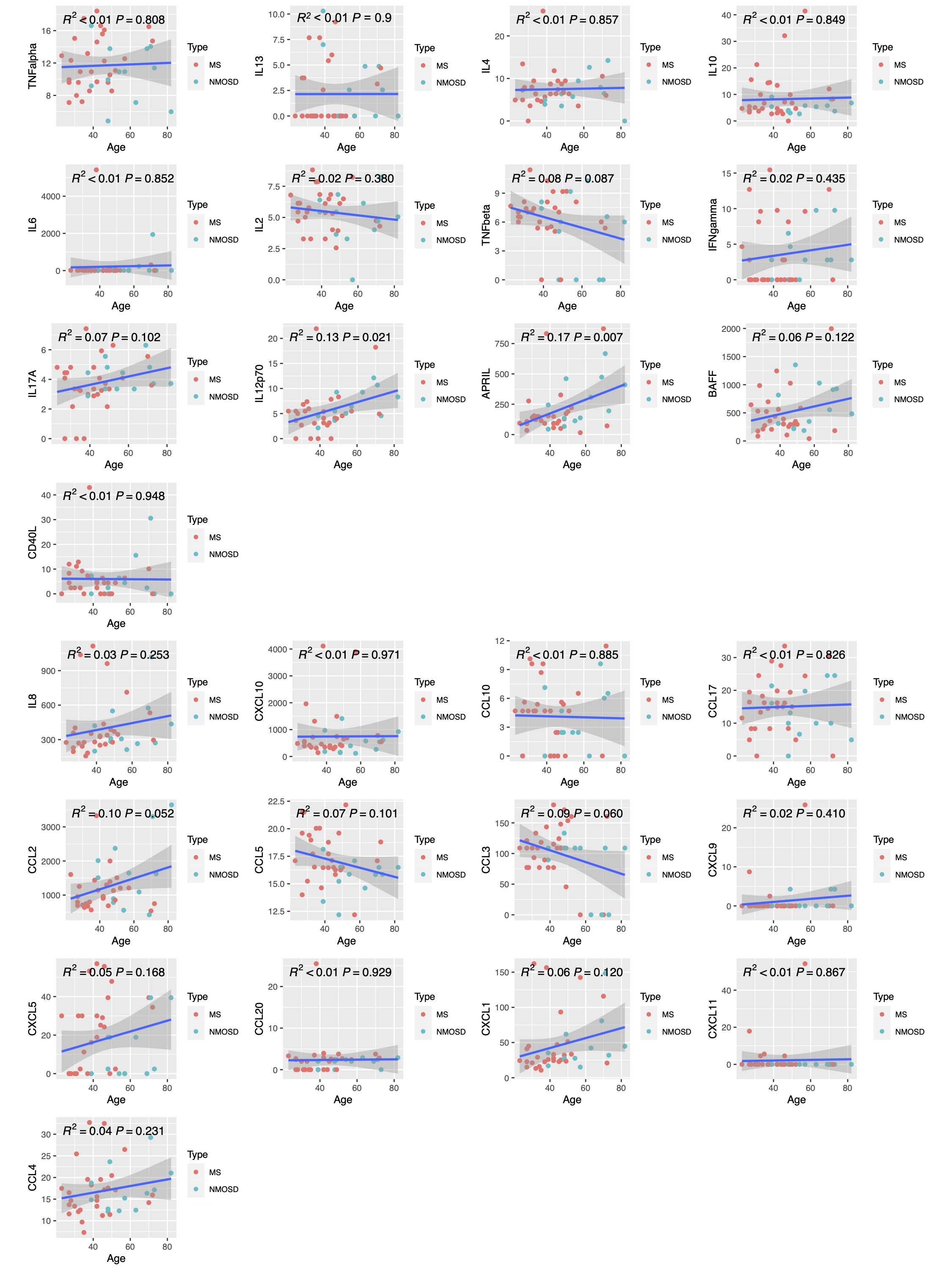

Supplement: Supplementary Figure 1 — Correlation of CSF inflammatory parameters with age at sample collection. Scatterplot graphs of the linear regression analysis showed the relation between cytokine/chemokine levels in CSF and age at sample collection. Strengths of the associations are given in R2 values. CSF, cerebrospinal fluid; MS, multiple sclerosis; NMOSD, neuromyelitis optica spectrum disorder. [file Image_1.JPEG]

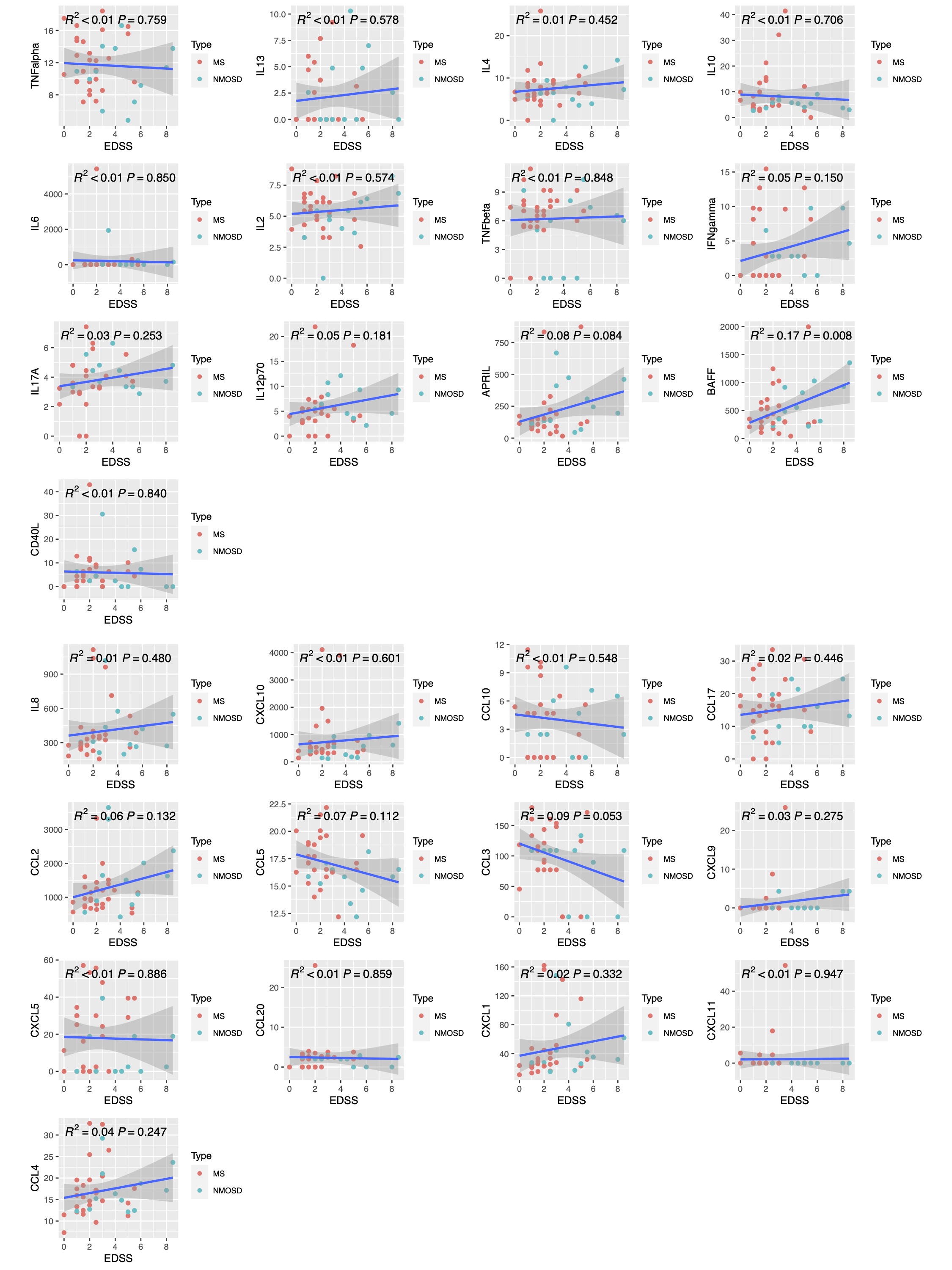

Supplement: Supplementary Figure 2 — Correlation of CSF inflammatory parameters with EDSS at sample collection. Scatterplot graphs of the linear regression analysis showed the relation between cytokine/chemokine levels in CSF and EDSS at sample collection. Strengths of the associations are given in R2 values. CSF, cerebrospinal fluid; MS, multiple sclerosis; NMOSD, neuromyelitis optica spectrum disorder; EDSS, Expanded Disability Status Scale. [file Image_2.JPEG]

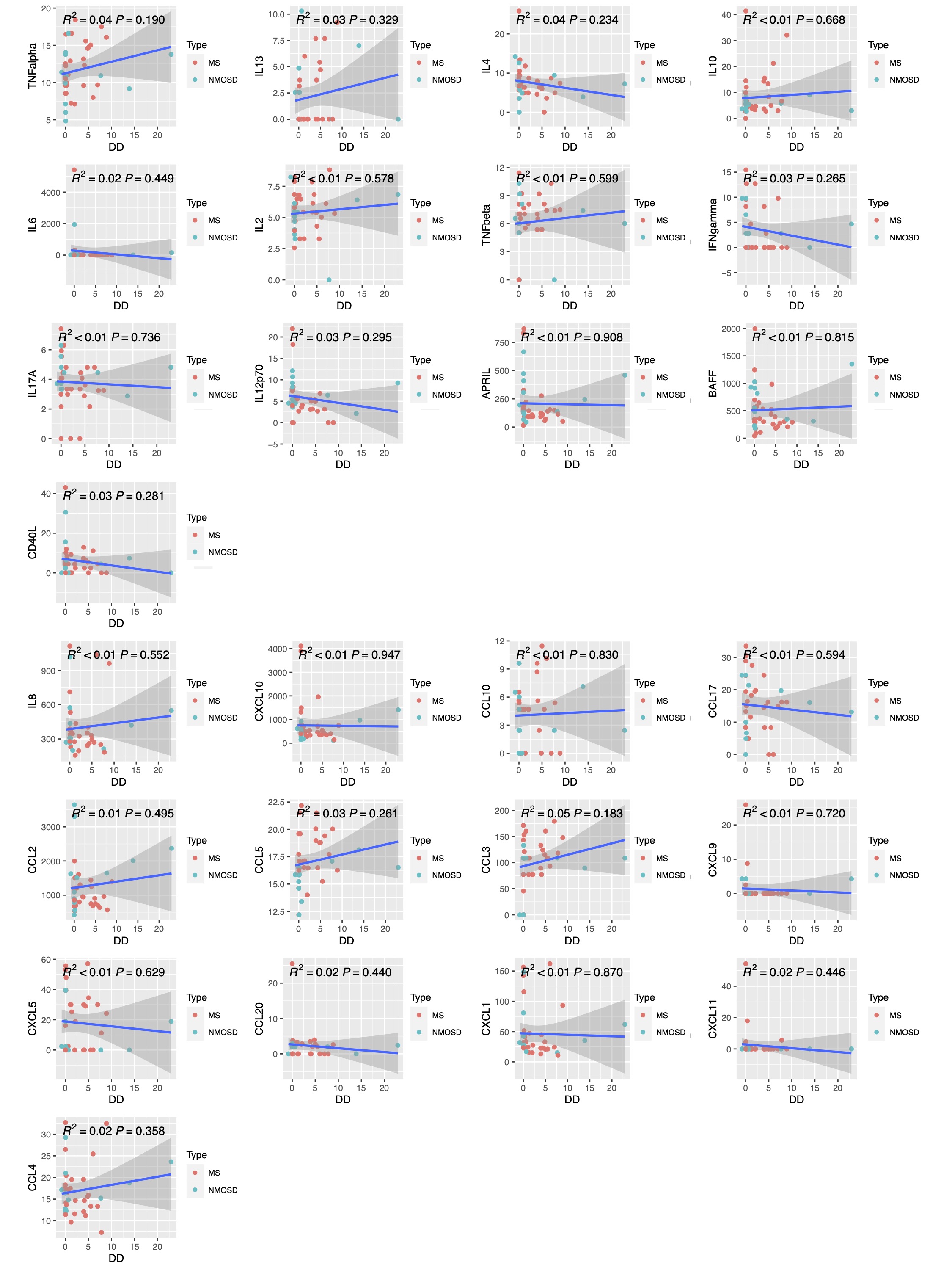

Supplement: Supplementary Figure 3 — Correlation of CSF inflammatory parameters with disease duration at sample collection. Scatterplot graphs of the linear regression analysis showed the relation between cytokine/chemokine levels in CSF and DD at sample collection. Strengths of the associations are given in R2 values. The DD was defined as the term (year) from first episode with neurological deficit to sample collection. CSF, cerebrospinal fluid; MS, multiple sclerosis; NMOSD, neuromyelitis optica spectrum disorder; DD, disease duration. [file Image_3.JPEG]

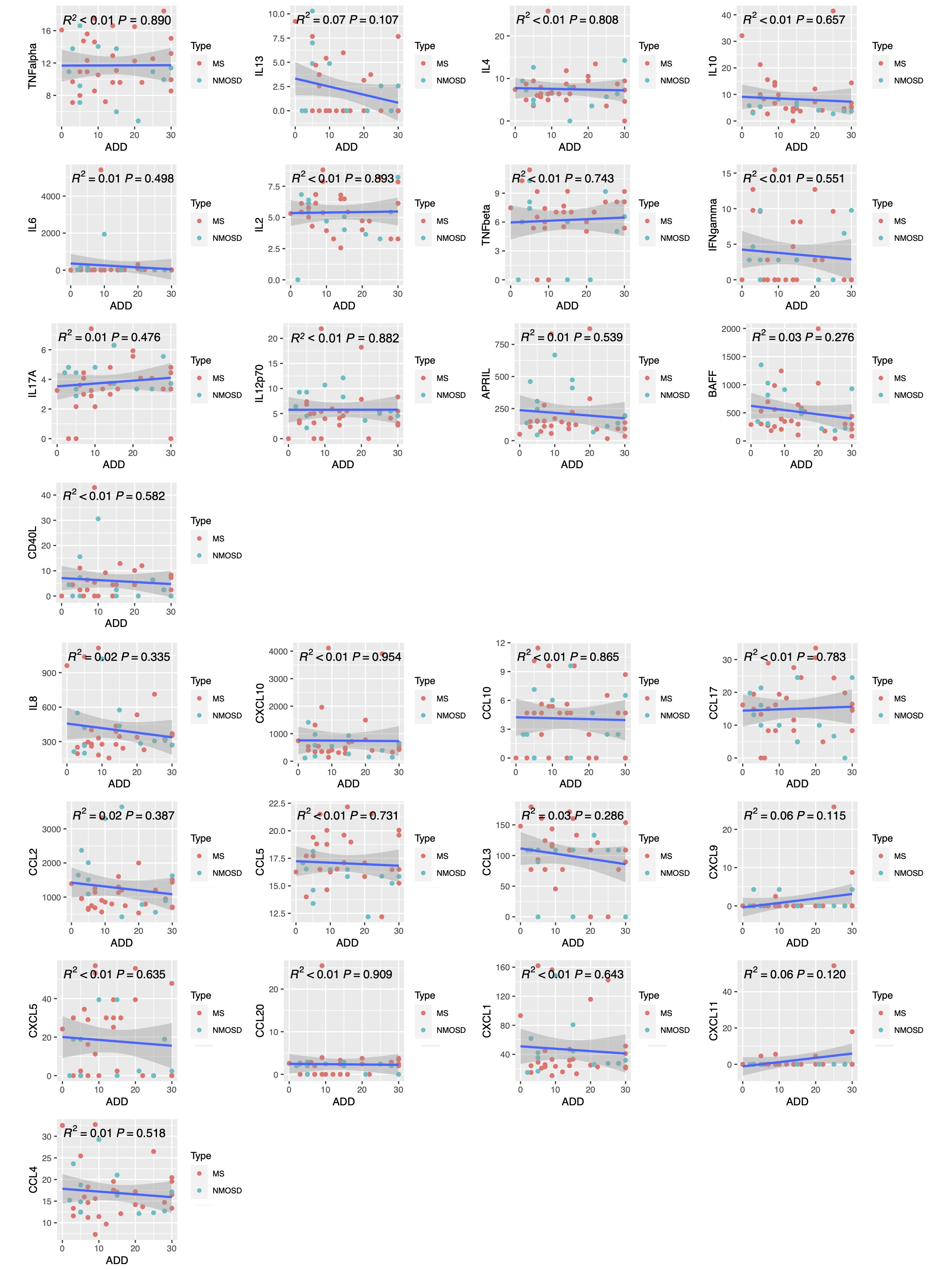

Supplement: Supplementary Figure 4 — Correlation of CSF inflammatory parameters with duration of acute exacerbation at sample collection. Scatterplot graphs of the linear regression analysis showed the relation between cytokine/chemokine levels in CSF and ADD at sample collection. Strengths of the associations are given in R2 values. The ADD was defined as the term (day) from onset of acute neurological deficit to sample collection. CSF, cerebrospinal fluid; MS, multiple sclerosis; NMOSD, neuromyelitis optica spectrum disorder; ADD, acute disease duration. [file Image_4.JPEG]
